# Laparoscopic splenectomy as a definitive management option for high-grade traumatic splenic injury when non operative management is not feasible or failed: a 5-year experience from a level one trauma center with minimally invasive surgery expertise

**DOI:** 10.1007/s13304-021-01045-z

**Published:** 2021-04-10

**Authors:** Arianna Birindelli, Matthew Martin, Mansoor Khan, Gaetano Gallo, Edoardo Segalini, Alice Gori, Amy Yetasook, Mauro Podda, Antonio Giuliani, Gregorio Tugnoli, Robert Lim, TraumaLap Study Group, Michael Cripps, Paschalis Gavriilidis, Antonio Affinita, Carlo Coniglio, Fausto Catena, Antonio Tarasconi, Belinda De Simone, Nicola De’ Angelis, Luca Ansaloni, Dario Tartaglia, Federico Coccolini, Massimo Chiarugi, Ferdinando Agresta, Gianluca Baiocchi, Gabriele Sganga, Isidoro Di Carlo, Francesco Pata, Marcelo Augusto Fontenelle Ribeiro, Daniel Souza Lima, Gustavo Pereira Fraga, Bruno Monteiro Pereira, Paolo Millo, Massimo Sartelli, Valeria Tonini, Maurizio Cervellera, Pierpaolo Sileri, Giovanni Del Vecchio, Pierluigi Marini, Salomone Di Saverio

**Affiliations:** 1 Department of Surgery, University of Bologna, Bologna, Italy; 2 General, Trauma and Emergency Surgery Unit, Bufalini Hospital, Cesena, Italy; 3 General and Emergency Surgery Unit, Esine General Hospital, ASST Valcamonica, Breno, BS Italy; 4 Department of Trauma and Acute Care Surgery, Scripps Mercy Hospital, San Diego, CA USA; 5 Digestive Diseases Department, Brighton and Sussex University Hospitals NHS Trust, Brighton, UK; 6 University of Magna Grecia, Catanzaro, Italy; 7 Department of General and Emergency Surgery, ASST, Crema, CR Italy; 8 Harbor-UCLA Department of Surgery, Torrence, CA USA; 9 Emergency and Minimally Invasive Surgery, Cagliari University Hospital, Cagliari, Italy; 10 General and Emergency Surgery Unit, Azienda Ospedaliera Regionale San Carlo, Potenza, Italy; 11 Trauma Surgery Unit, Emergency Department, Maggiore Hospital, Bologna, Italy; 12 Department of Surgery, Tripler Army Medical Center, Tripler, Honolulu, HI USA; 13 Cambridge Colorectal Unit, Cambridge University Hospitals NHS Foundation Trust, Addenbrooke’s Hospital, Cambridge Biomedical Campus, Hills Road, Cambridge, UK; 14 General Surgery Unit 1, Department of General Surgery, Ospedale Di Circolo, University of Insubria, ASST Sette Laghi, Regione Lombardia, Varese, Italy

**Keywords:** Abdominal trauma, Blunt abdominal trauma, Penetrating abdominal trauma, Trauma laparoscopy, Minimally invasive trauma surgery, Hemodynamic stability, Trauma surgery, Trauma center, Acute care surgery, Emergency laparoscopy, Laparoscopic splenectomy, Angio-embolization, Non-operative management

## Abstract

Technique, indications and outcomes of laparoscopic splenectomy in stable trauma patients have not been well described yet. All hemodynamically non-compromised abdominal trauma patients who underwent splenectomy from 1/2013 to 12/2017 at our Level 1 trauma center were included. Demographic and clinical data were collected and analysed with per-protocol and an intention-to-treat comparison between open vs laparoscopic groups. 49 splenectomies were performed (16 laparoscopic, 33 open). Among the laparoscopic group, 81% were successfully completed laparoscopically. Laparoscopy was associated with a higher incidence of concomitant surgical procedures (*p* 0.016), longer operative times, but a significantly faster return of bowel function and oral diet without reoperations. No significant differences were demonstrated in morbidity, mortality, length of stay, or long-term complications, although laparoscopic had lower surgical site infection (0 vs 21%).The isolated splenic injury sub-analysis included 25 splenectomies,76% (19) open and 24% (6) laparoscopic and confirmed reduction in post-operative morbidity (40 vs 57%), blood transfusion (0 vs 48%), ICU admission (20 vs 57%) and overall LOS (7 vs 9 days) in the laparoscopic group. Laparoscopic splenectomy is a safe and effective technique for hemodynamically stable patients with splenic trauma and may represent an advantageous alternative to open splenectomy in terms of post-operative recovery and morbidity.

**Supplementary Information:**

The online version contains supplementary material available at 10.1007/s13304-021-01045-z.

## Introduction

The spleen is the most commonly injured solid organ in abdominal trauma, and the most commonly injured structure in the abdomen following blunt trauma [1]. Despite the extensive use of non-operative management (NOM) and newer adjuncts such as angioembolization (AE) for hemodynamically non-compromized patients with splenic injuries even in high-grade injuries [2], splenectomy in hemodynamically non-compromized or ‘quasi-stable’ patients continues to play an important role in trauma surgery [3].

Although there are clear benefits to NOM (with or without AE) in terms of avoidance of splenectomy and complications associated with a laparotomy, there are many potential negative factors associated with NOM. These include its costs and morbidities, it is not always technically feasible or successful, it requires a strict patient conduct and close expert monitoring, it does not completely prevent delayed splenic rupture or hemorrhage, and it requires the immediate availability of an operating room and operative team at all times [4].

Indications for splenectomy in hemodynamically non-compromised patients include blunt or penetrating splenic injury requiring surgical exploration for diaphragmatic or hollow-viscus injuries, high-grade blunt splenic injury with unavailable, contraindicated, unfeasible or unsuccessful NOM and AE and all complications following AE such as pseudoaneurysms, splenic infarction or abscess, and delayed rupture. NOM is contraindicated in patients unable or unwilling to comply with the strict NOM conduct and activity restrictions (e.g., mentally impaired, homeless, self-employed, professional athletes), as well as those with an unreliable examination typically due to associated injuries and intubation [5–9]. There are also contraindications to AE as an adjunct, including patients severely allergic to intravenous iodine contrast or with late stage chronic kidney disease. AE may also be unfeasible because of a non-cooperative patient or due to technical reasons, such as celiac trunk stenosis, tortuous and kinking splenic artery, or failure in releasing the coils. In some cases, even if AE is technically performed, it can fail to achieve hemostasis or cause splenic infarction and abscess formation that requires percutaneous interventional or operative interventional. Furthermore, due to the high risk of NOM failure (80%), some authors recommend splenectomy in grade IV splenic injuries with sub capsular hematoma or vascular abnormalities (pseudoaneurysms) [10]. Splenectomy may be performed in patients with blunt splenic injury and multiple severe skeletal injuries requiring prolonged and invasive orthopaedic procedures in a prone position to avoid the risk of simultaneous bleeding from multiple sites. According to the surgeon’s evaluation, splenectomy may also be indicated in patients affected by splenomegaly (e.g., lymphoma, tropical, auto-immunity, portal hypertension [6, 11]) or in any case the trauma surgeon deems NOM and AE not safe or indicated. Table 1 summarizes the possible indications for splenectomy in hemodynamically non-compromised trauma patients.

**Table 1:** Indications for splenectomy in hemodynamically stable trauma

High–moderate grade (AAST grade II and above) splenic injury and clinical or radiological findings suggestive of a possible traumatic hollow viscus injury, diaphragmatic injury or other abdominal source of hemorrhage not amenable by AE (e.g., mesenteric injury)
High–moderate grade (AAST grade II and above) splenic injury with blush in penetrating trauma
High–moderate grade (AAST grade II and above) splenic injury with subcapsular hematoma
High–moderate grade (AAST grade II and above) splenic injury (with or without blush) and NOM ± AE contraindicated (e.g., pregnant, mentally impaired, homeless, severe allergy to intravenous iodine contrast, kidney failure)
High–moderate grade (AAST grade II and above) splenic injury (with or without blush) and NOM ± AE refused by patient (e.g., self-employed, professional athletes)
High–moderate grade (AAST grade II and above) splenic injury with blush and NOM with AE technically not feasible (e.g., tortuous splenic artery, celiac trunk stenosis, failure in releasing the coils)
High–moderate grade (AAST grade II and above) splenic injury (with or without blush) and NOM ± AE failed (e.g., persistent blush or persistent venous oozing, pseudoaneurisms, delayed-rupture, splenic abscess)
High–moderate grade (AAST grade II and above) splenic injury with blush NOM and AE unavailable
Splenic injury without blush and with significant haemoglobin drop due to persistent venous oozing
High-grade (AAST grade III and above) splenic injury (with or without blush) and need of urgent complex orthopaedic surgery (especially if in prone position, e.g., spinal and pelvic surgery)
High–moderate grade (AAST grade II and above) splenic injury in pathologic splenomegaly (e.g., lymphoma, tropical, autoimmunity, portal hypertension)

As a large proportion of the morbidity associated with standard open splenectomy is attributed to the incision (pain, respiratory distress, wound infection, incisional hernia) and the development of adhesions from open bowel manipulation, the use of minimally invasive techniques represents an intriguing potential option to treat the splenic injury while avoiding many of the risks associated with open surgery. In addition, minimally invasive surgery allows for a faster post-operative recovery and shorter length of stay (LOS) compared to open surgery [12, 13]. Therefore, laparoscopic splenectomy (LS) may be an advantageous alternative to open splenectomy (OS), either as an early or delayed procedure for splenic trauma, failure of nonoperative management, or treatment of AE associated complications [14, 15]. Advantages and disadvantages of NOM and LS are listed in Table 2. Nevertheless, LS in hemodynamically non-compromised patients with splenic injuries is not widely accepted yet and only a few case reports and small case-series have been published in the literature [16–25].

**Table 2:** Advantages and disadvantages of non-operative management (NOM) and laparoscopic splenectomy (LS)

	Advantages	Disadvantages
NOM	Preserve spleen function Avoid surgery	Strict conduct for many weeks Long LOS High radiation exposure Long time to return to work and daily life High morbidity and potential mortality associated with failures High cost (angio-suite, angiography devices and coils, follow-up) Risk of delayed rupture
LS	Short LOS and fast recovery without strict conduct in case of isolated splenic injury Fast return to work and daily life Minimal invasiveness Small scars	Loss of splenic function (immunologic and hematologic), vaccine required, increase of platelets count Surgical intervention

In this article we sought to analyze our institutional experience with LS for splenic trauma in hemodynamically non-compromised patients. Our primary objectives were to investigate the indications, safety, feasibility, and outcomes of LS in this group of patients. In addition, we sought to outline key tips and tricks for performing LS for trauma, as well as describing the step-by-step surgical technique and an associated procedural video.

## Materials and methods

This is a retrospective observational study including all hemodynamically non-compromised (ATLS class I or *II–III responders)* trauma patients who underwent splenectomy between January 2013 and December 2017 at the Level 1 Trauma Center of the Maggiore Hospital in Bologna (Italy). The study was reviewed and approved by the institutional review board. Written consent was collected for all patients prior to surgery.

All patients underwent contrast-enhanced computed tomography (CE CT-scan) on admission as part of their radiologic trauma evaluation. Decision to consider NOM not feasible or failed and indications for a definitive splenectomy was made by experienced trauma surgeons in a multidisciplinary discussion with Trauma ICU and Interventional Radiology attendings (Consultants).

All patients were hemodynamically stable and fully resuscitated, and all had a High-Grade (≥ 3) splenic injury. Selection between the open or laparoscopic approach was made randomly and exclusively based upon the calendar of the on call roster and the availability on duty of an Attending Trauma Surgeon with advanced minimally invasive surgery (MIS) expertise. Whenever the on call Attending Trauma Surgeon was a surgeon without advanced MIS expertise, the splenectomy was made via a traditional open midline laparotomy. Whenever was on duty the on call attending experienced trauma surgeon with advanced laparoscopic skills and a completed formal training (fellowship or above) in MIS (SDS fulfilled these criteria in our institution), the splenectomy was approached/performed laparoscopically.

In either case, the criteria for the Decision to consider NOM not feasible or failed and indications for a definitive splenectomy did not differ and was made by experienced trauma surgeons in a multidisciplinary discussion with Trauma ICU attending and Interventional Radiology Attendings. Selection of the open or laparoscopic approach between the 2 group was made exclusively based upon the calendar of the on call roster and the availability on duty of an Attending Trauma Surgeon with advanced MIS expertise.

Exclusion criteria for enrollment in the study were:

• Hemodynamic instability transiently or not responding/refractory to resuscitation
• Septic shock
• Evidence of severe retroperitoneal organ injury at CT scan requiring surgical exploration
• Contraindications to pneumoperitoneum (severe head injury, cardio-respiratory failure)

*Early* procedure was defined as a surgical operation performed within 24 h from admission, while this was defined as *delayed* if performed after 24 h from admission.

Failed splenic NOM (with or without AE) was defined as:

• Clinical or radiological findings suggestive of a possible hollow viscus injury or other abdominal source of hemorrhage:
• Delayed splenic rupture (a significant hemorrhage from a ruptured spleen more than 48 h after injury)
• Technical failure of AE (e.g., tortuous splenic artery, celiac trunk stenosis, failure in releasing the coils)
• Complications of AE requiring operative intervention (e.g., persistent blush or persistent venous oozing, abscess, pseudoaneurysm)

### Surgical technique of LS

**Fig. 1:**
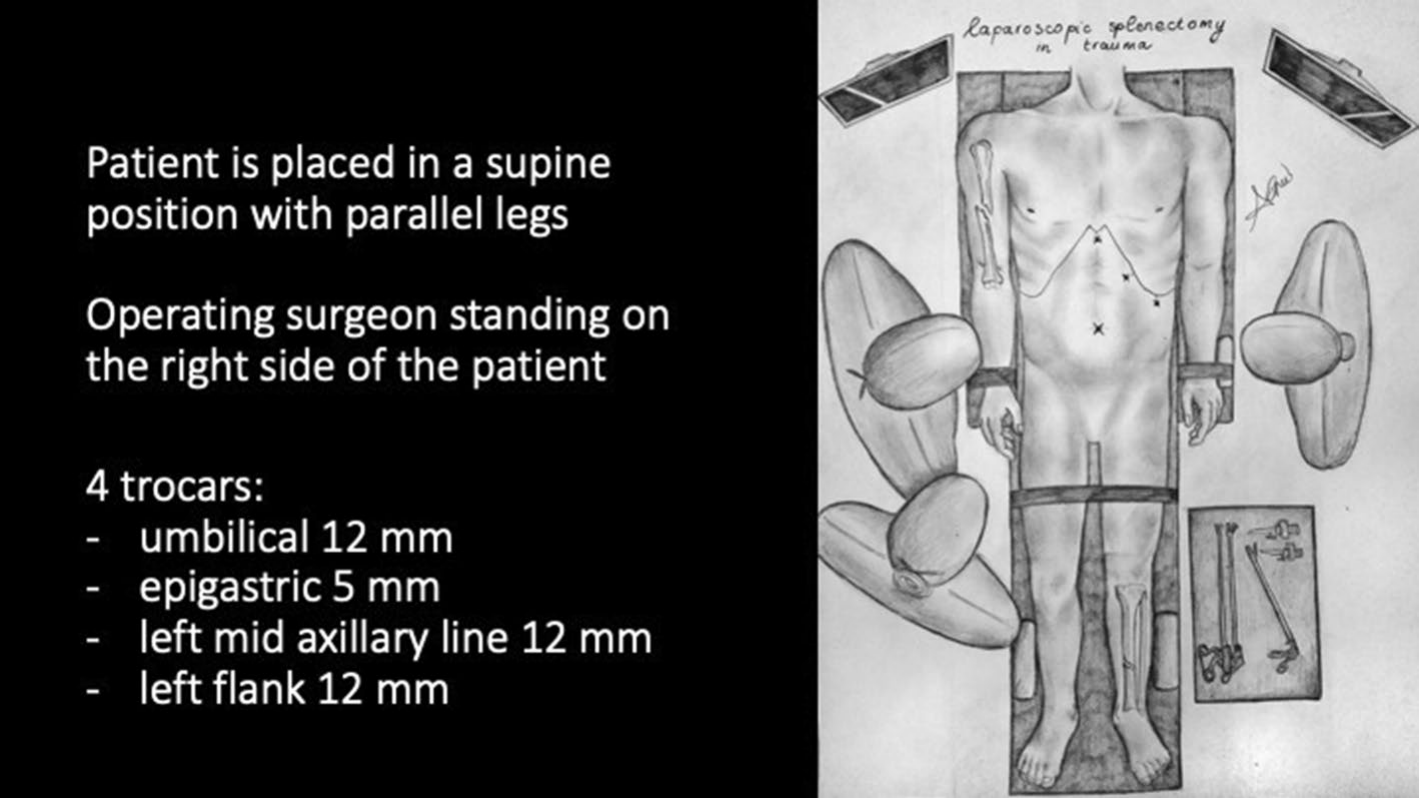
Patient and trocar position

The patient is placed in a supine position with straight and parallel legs with surgeons standing on the right side of the patient, particularly in cases with associated complex orthopedic or pelvic injuries (Fig. 1). Although some surgeons prefer standing between the legs in a low lithotomy position or using a split-leg table to better access the left upper quadrant (LUQ), we feel this is contraindicated in cases of blunt trauma with concomitant lower spinal, pelvic, and/or lower extremity orthopedic fractures. We also feel that this offers little to no technical advantage to the performance of LS vs the operating surgeon standing on the patient's right side. In the absence of scapular or vertebral fractures or severe left thoracic trauma, a pillow or bump under the left hemi-thorax or left scapula can be useful to better expose the left upper quadrant and use gravity to displace the spleen medially. After securing the patient to the table with belts, a moderate left-side up tilt and reverse-Trendelenburg position can be achieved.

**Fig. 2:**
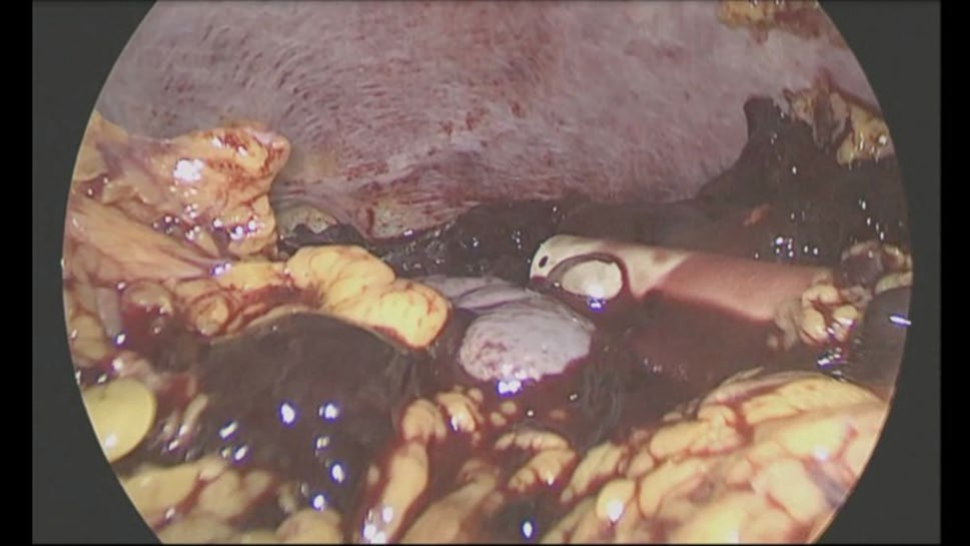
Minimal evacuation of clots and free fluid

**Fig. 3:**
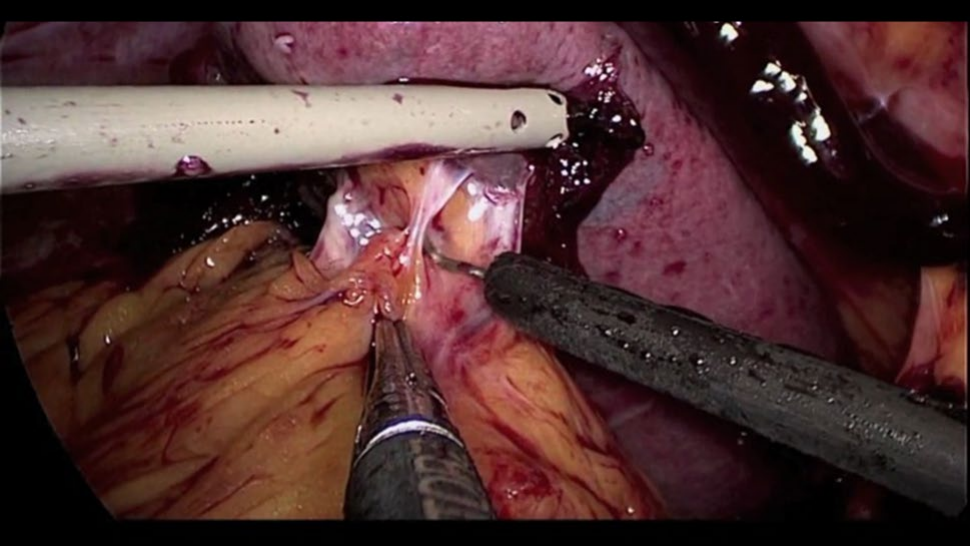
Spleen exposition and spleno-colic ligament division

**Fig. 4:**
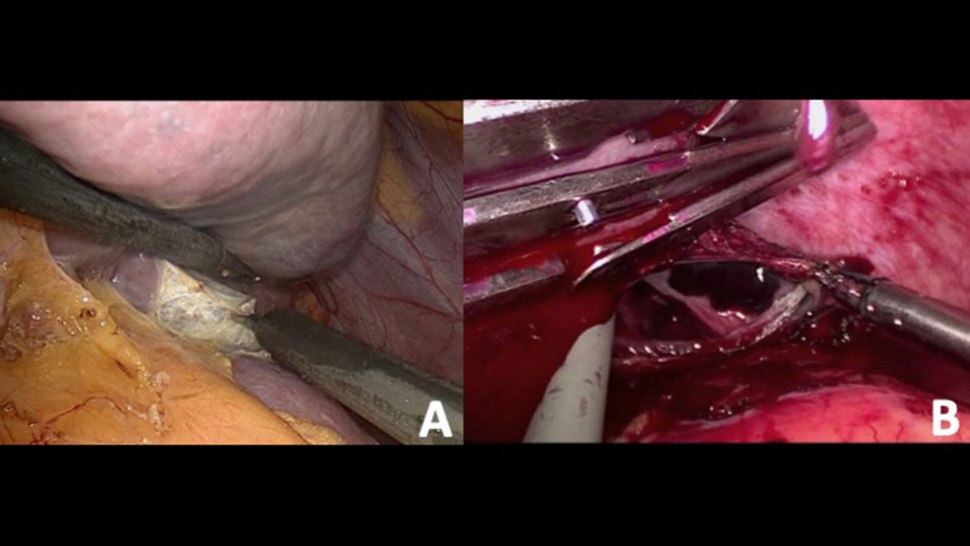
Spleno-renal ligament division in a case of blunt splenic injury with subcapsular hematoma (**a**) and in a case of splenic parenchymal tear (**b**)

After umbilical open access pneumoperitoneum is gradually induced, 3 additional trocars are placed under direct vision, 2 in the left flank and 1 in the epigastrium, so that the incisions can be connected in case of conversion to open (Fig. 1). Minimal evacuation of the blood clots in the LUQ is recommended to avoid resumption of active bleeding if hemostasis has been achieved spontaneously (Fig. 2). The splenic inferior pole is bluntly retracted cephalad (e.g., with suction tube) to expose the spleno-colic ligament and the splenic inferior polar vessels. The first one is divided by monopolar hook, while the second can be stapled, sealed or ligated with clips and divided (Fig. 3). The spleen is then mobilized medially using primarily blunt dissection to expose the posterior attachments (spleno-renal ligament). It is important to note that in blunt splenic trauma, many of these ligamentous attachments have already been torn by the blunt force trauma. The remaining posterior attachments are then divided both bluntly and with the monopolar hook, taking care to avoid injuring the hilar vessels which should now come into view (Fig. 4).

**Fig. 5:**
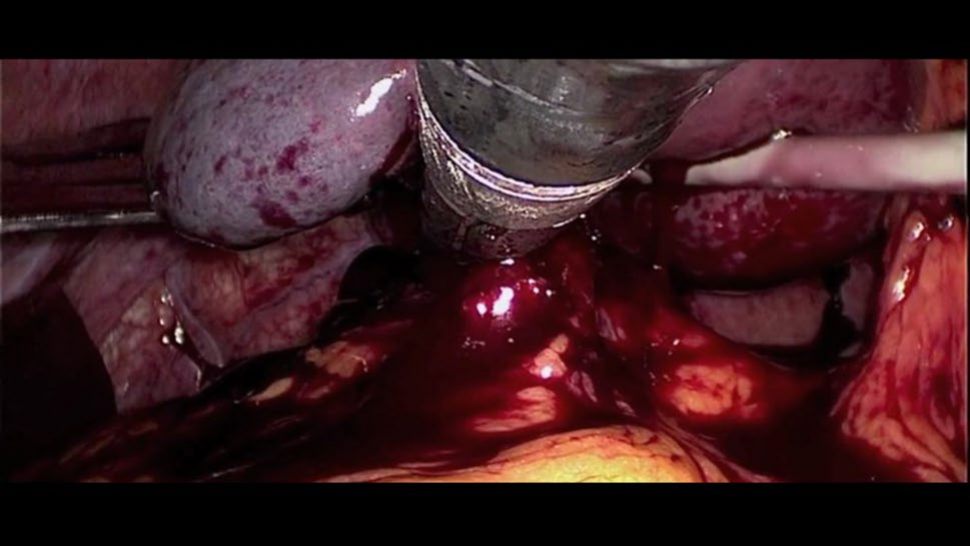
Hanging manoeuvre and splenic hilum stapling

**Fig. 6:**
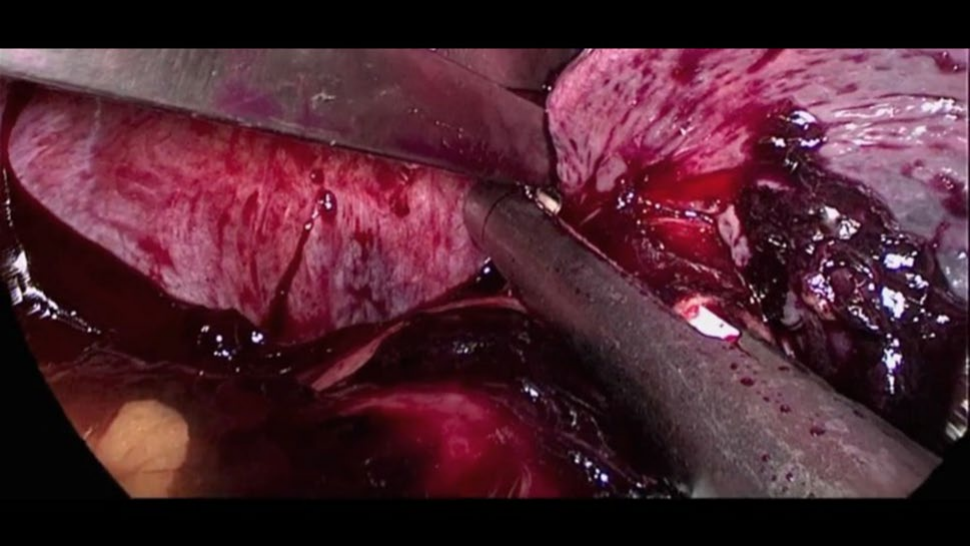
Short gastric vessels stapling and spleno-diaphragmatic ligament division

**Fig. 7:**
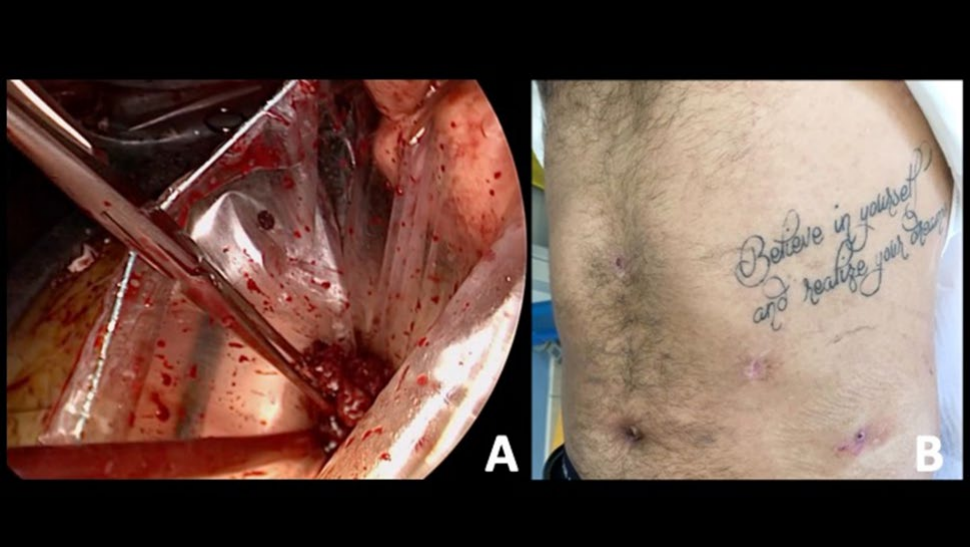
Specimen retrieval, morcellation through the umbilical access (**a**) and functional outcome (**b**)

**Fig. 8:**
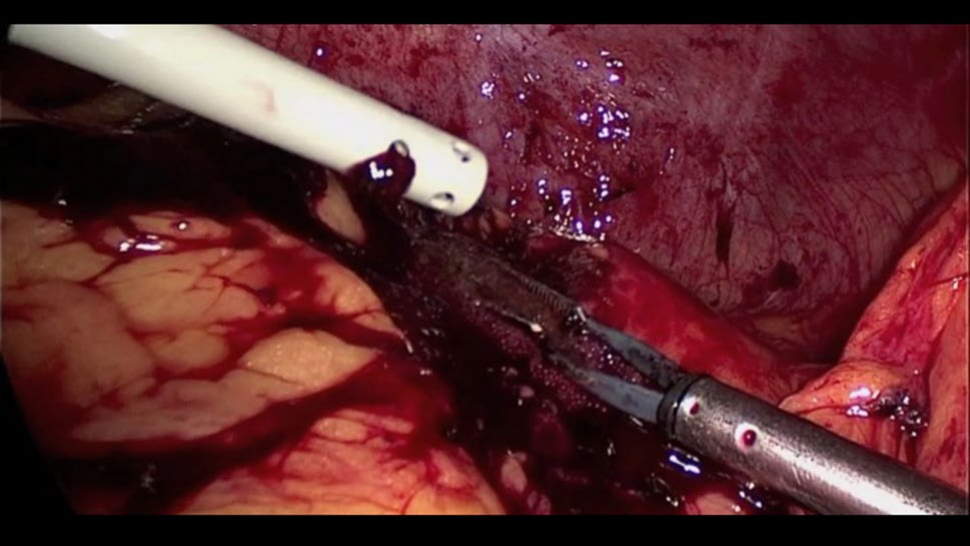
Abdominal washout and hemostasis

The splenic hilum is then safely exposed by bluntly suspending the spleen upwards with 2 instruments, 1 in the medial and the other in the lateral side (e.g., suction tube and endoretractor), to avoid further parenchymal trauma and capsular tears, which may worsen intra-operative bleeding from the injured splenic parenchyma. At this point it is critical to examine the hilar area to both identify the splenic artery and vein structures, as well as ensure that the tail of the pancreas is uninjured and is mobilized away from the planned transection point on the hilum. The hilum is then clamped and divided with a vascular-cartridge flexible endoscopic stapler, taking care to ensure there is no stomach, pancreas, or colon caught in the jaws prior to firing (Fig. 5). The short gastric vessels are stapled and divided with a separate load, or can be divided/sealed with an energy device. A key technical point is to leave the superior attachments of the spleen to the diaphragm (spleno-diaphragmatic ligament) as the last intact structure. This so-called “hanging spleen technique” helps to optimize the exposure of the key medial, lateral, and inferior structures and avoids having a floppy spleen that falls on top of the hilar structures precluding safe dissection. The spleno-diaphragmatic ligament is then divided with hook cautery taking care to avoid injuring the diaphragm, and thus the splenectomy has been safely completed (Fig. 6). The resected spleen is retrieved in an endoscopic retrieval bag, completely morcellated and extracted through the umbilical access (Fig. 7). After suction evacuation of blood clots and irrigation of the left upper quadrant, hemostasis of the splenic bed must be verified and eventually completed with aid of bipolar energy and topical hemostatic agents. It is particularly important to carefully search for any avulsed short gastric vessels along the proximal greater curve of the stomach which may cause delayed hemorrhage if not identified and controlled during the index procedure. Careful exploration of the left diaphragm, splenic colonic flexure, and gastric greater curvature to exclude associated injuries is recommended. It is also important to do a thorough search of the left upper quadrant and remainder of the abdomen to identify and remove any splenic fragments as can be seen with high-grade injuries (Fig. 8). Corrugated drains are inserted in the left upper quadrant and lesser sac area if needed. The full step-by-step surgical procedure is shown in Video 1.To watch the video attached to the manuscript (MP4 1239012 KB)

In case of splenic abscess after AE and large purulent collections, we advise to bluntly divide any inflammatory adhesions between the spleen, omentum, and colon using the laparoscopic suction/irrigator device, which we feel is the safest way to develop an avascular plane. Perisplenic purulent collections are entered bluntly and drained. Capsular tears can be controlled with bipolar coagulation at high settings and gauze or topical hemostatic packing. The rest of the procedure is the same as previously described. For penetrating trauma involving the spleen, the principles of performing a LS are identical to that described above. In addition to the splenectomy, care should be taken to carefully examine the entire diaphragm for any laceration and to perform a suture repair as needed. Rapid open conversion is warranted in case of sudden hemodynamic instability, unexpected and uncontrollable bleeding or inability to expose or completely control the splenic hilum.

### Data collection

Baseline demographic and clinical data were recorded including: age, gender, comorbidities, date and mechanism of trauma, admission date and vital signs, laboratory and imaging results, indication to surgical intervention, pre-operative vital signs, date and type of surgery, conversion rate, operating time, intra- and post-operative blood transfusion, length of stay in intensive care unit (ICU), post-operative gastrointestinal recovery [nasogastric tube (NGT) removal, feeding, bowel function], morbidity, mortality and overall length of stay. In addition to data from the index admission, all patients underwent a structured telephone follow-up in May 2018 to assess the rate of long-term complications such as incisional hernia and symptoms of small bowel obstruction.

### Statistical analysis

Qualitative and quantitative data were descriptively analyzed. For each patient American Society of Anesthesiologists (ASA) score, American Association for the Surgery of Trauma (AAST) splenic injury grade, and Injury Severity Score (ISS) were calculated. AAST grade was assigned by an experienced and dedicated radiologist based on the CT images. ISS was stratified as follows: 1–8 minor, 9–15 moderate, 16–25 severe and > 25 very severe.

Descriptive statistics data were expressed as fraction (percentage), mean, median and range (minimum–maximum). Results were analyzed using chi-square test and Fisher’s exact test, as appropriate, for proportions in case of discrete data. Continuous data was analyzed using the independent samples *t* test. A *p* value of < 0.05 was considered statistically significant. A *per-protocol* and an *intention-to-treat* analysis comparing the splenectomies performed with a minimally invasive technique (LS) and those performed with a traditional open laparotomy (OS) in the same time period were carried out. Finally, to reduce the bias due to associated injuries and other surgical procedures, subgroup analyses of patients with isolated splenic injuries were performed. The primary outcome of the study was to evaluate the feasibility and safety of LS in stable patients with abdominal trauma as a non-inferior approach to traditional OS. Secondary outcomes analysed were the effect of laparoscopy on post-operative recovery in terms of bowel function and short- and long-term complications.

## Results

Between January 2013 and December 2017, 48 hemodynamically stable patients underwent splenectomy for splenic trauma at our institution: 32 procedures (67%) were OS, while 16 of them (33%) were approached in a minimally invasive manner (LS), with a conversion rate of 19% (3/16) (Table 3). All but 1 patient were blunt trauma.

**Table 3:** Intention-to-treat analysis between open (OS) and laparoscopic (LS) splenectomies

Characteristics		OS [32]	LS [15]	*p*
Gender	M	22	13	0.497
	F	10	3	
Age (years)	Average	50	48	0.359
	Median	49	44	
	Range	21–86	24–88	
ASA	I	17	6	0.633
	II	11	7	
	III	3	3	
	IV	1	0	
Mechanism of trauma	Blunt	32	15	1
	Penetrating	0	1	
Pre-operative vital signs	HR (bpm) average	89	94	0.415
	Median	85	90	
	Range	60–130	70–130	
	SPB (mmHg) average	116	126	0.262
	Median	120	130	
	Range	70–150	95–160	
	GCS 15	27	16	1
	14	4	0	
	13	1	0	
	ATLS class I	14	11	0.095
	II-responder	16	4	
	III-responder	2	1	
Pre-operative blood test	BE (mmol/L) average	− 2	− 0.6	0.348
	Median	−1.3	− 0.7	
	Range	− 9–5.3	− 9.4–9.7	
	Lactate (mmol/L) average	2	1.5	0.306
	Median	1.4	1.3	
	Range	0.5–6.1	0.6–3,3	
	HB (g/dL) average	12	12.2	0.436
	Median	12.75	11.7	
	Range	5.3–15.6	8.2–19.1	
ISS	Average	20	23	0.259
	Range	4–57	1–57	
Timing	Early	23	7	0.140
	Delayed	9	9	
Failed NOM		10	8	0.206
AAST grade	I	1	1	0.288
	II	3	1	
	III	8	9	
	IV	13	5	
	V	7	0	
Associated injuries		13	9	0.366
Operative time (min)	Average	60	126	**< 0.00001**
	Median	55.5	124	
	Range	28–106	77–193	
Other surgical procedures		5	8	**0.016**
Blood trasfusions	Intre-operative	16	4	0.128
	Post.operative	8	9	0.054
Intensive care unit	Admission	20	7	0.237
	Average length of stay (days)	3.5	9	**0.042**
Bowel function recovery (average POD)	NGT removal	2.7	1	**< 0.00001**
	Feeding	3.8	1	**< 0.00001**
	Flatus	3.6	3	**0.037**
	Stool	5.9	5	**0.035**
Morbidity	Overall	22	12	0.746
	Re-operation	2	0	0.546
	SSI	7	0	0.079
Mortality		1	1	1
Overall length of stay (days)	Average	18	20	0.455
Follow-up	Incisional hernia	6	2	0.701
	Bowel obstruction	3	0	0.541

Both the *intention-to-treat and per-protocol* analyses (Tables 3, 4) of the overall population did not show significant differences between the groups baseline characteristics in terms of age, vital signs, laboratory values, ASA score, ISS, AAST injury grade, and associated injuries. NOM with AE had an overall failure rate of 50% among the 16 patients in the LS group. This included one early AE failure (unfeasible due to celiac trunk stenosis) and 7 cases with initial success of AE at haemorrhage control but then later failure due the development of pseudoaneurisms in 4 patients, oozing in 2 patients and splenic abscess in 1 patient. NOM with AE had been successfully performed but later failed in 10 out of 32 cases in the OS group (31%) (oozing in 5 patients, delayed rupture in three patients, splenic infarction in 2 patients). Indications for splenectomy were decided upon the multidisciplinary evaluation but also by the on-call trauma surgeon given his/her evaluation, experience and discretion upon clinical evaluation, imaging characteristics, and laboratory values at presentation and over time (Table 5). Early procedures were more common in the OS group (71%, 23/32), while delayed procedures were more common in the LS group (56%, 9/16). The most delayed procedure was 15 days from trauma. The average delay was 6 days in the open group and 2 days in the laparoscopic group. In examining the operative and post-operative outcomes, there were several statistically significant differences in favour of LS. These included an increased incidence of associated surgical procedures performed (*p* 0.016) and a significantly shorter time to bowel function recovery compared to OS (*p* < 0.0001). The operating time (*p* < 0.0001) and ICU length of stay (*p* 0.042) were significantly higher in the LS group, but these differences appeared to be mainly attributed to the increased incidence of associated procedures in the LS group and not to the splenectomy itself. The 2 techniques were comparable in terms of morbidity, mortality, overall LOS and long-term complications. Since a significantly higher rate of associated surgical procedures were found in the LS group, further *intention-to-treat and per-protocol* analyses for the subgroup of isolated splenic injuries were performed (Tables 6, 7). This sub-analysis demonstrated no significant difference in ICU length of stay between the 2 groups and demonstrated similar findings as described for the entire cohort. Of note, although operative time with LS was significantly longer vs OS, the laparoscopic approach was associated with a significantly faster recovery in terms of time to NGT removal, time to initiate diet, and time to first bowel movement.

**Table 4:** Per-protocol comparison between open (OS) and laparoscopic (LS) splenectomies

Characteristics		OS [32]	LS [12]	*p*
Gender	M	22	10	0.725
	F	10	3	
Age (years)	Average	50	47	0.458
	Median	49	41	
	Range	21–86	24–88	
ASA	I	17	5	0.709
	II	11	6	
	III	3	2	
	IV	1	0	
Mechanism of trauma	Blunt	32	12	1
	Penetrating	0	1	
Pre-operative vital signs	HR (bpm) average	89	93	0.315
	Median	85	90	
	Range	60–130	70–120	
	SPB (mmHg) average	116	126	0.086
	Median	120	125	
	Range	70–150	95–160	
	GCS 15	27	13	1
	14	4	0	
	13	1	0	
	ATLS class I	14	7	0.247
	II-responder	16	2	
	III-responder	2	1	
Pre-operative blood test	BE (mmol/L) average	− 2	− 1.1	0.448
	Median	− 1.3	− 0.1	
	Range	− 9–5.3	− 9.4–3.2	
	Lactate (mmol/L) average	2	1.37	0.279
	Median	1.4	1.2	
	Range	0.5–6.1	0.6–3.3	
	HB (g/dL) average	12	12.6	0.252
	Median	12.75	12.3	
	Range	5.3–15.6	8.2–19.1	
ISS	Average	20	24	0.177
	Range	4–57	1–57	
Timing	Early	23	6	0.169
	Delayed	9	7	
Failed NOM		10	7	0.156
AAST grade	I	1	1	0.235
	II	3	1	
	III	8	8	
	IV	13	3	
	V	7	0	
Associated injuries		13	8	0.323
Operative time (mins)	Average	60	128	**< 0.00001**
	Median	55.5	129	
	Range	28–106	83–193	
Other surgical procedures		5	7	**0.021**
Blood trasfusions	Intra-operative	16	3	0.182
	Postoperative	8	6	0.286
Intensive care unit	Admission	20	6	0.341
	Average length of stay (days)	3.5	10	**0.049**
Bowel function recovery (average POD)	NGT removal	2.7	1	**< 0.00001**
	Feeding	3.8	2	**< 0.00001**
	Flatus	3.6	3	0.107
	Stool	5.9	5	0.089
Morbidity	Overall	22	9	1
	Re-operation	2	0	0.546
	SSI	7	0	0.089
Mortality		1	1	1
Overall length of stay (days)	Average	18	20	0.458
Follow-up	Incisional hernia	6	2	1
	Bowel obstruction	3	0	0.546

**Table 5:** Indications to splenectomy (multiple indications are possible for each case)—open splenectomy (OS), laparoscopic splenectomy (LS)

Indication to splenectomy (each patients may have more than one indication for splenectomy)	OS	LS
Early		
Subcapsular hematoma	0	2
Urgent spinal, pelvic or complex orthopedic surgery	1	6
Mentally impaired patient	0	2
Celiac trunk stenosis	0	1
Penetrating splenic injury	0	1
Splenomegaly	1	2
Diaphragmatic injury	2	0
Delayed		
Pseudoaneurisms after AE	0	4
Persistent oozing (with or without AE)	5	2
Splenic infarction/abscess after AE	2	1
Delayed splenic ruwpture	3	0
On call surgeon’s own decision (surgeon’s preference/lap experience)	17	0

**Table 6:** Intention-to-treat analysis between open (OS) and laparoscopic splenectomy (LS) for isolated splenic injuries

Characteristics		OS [18]	LS [6]	*p*
Gender	M	11	5	0.664
	F	8	1	
Age (years)	Average	55	45	0.151
	Median	51	48	
	Range	21–86	24–59	
ASA	I	10	1	0.164
	II	6	2	
	III	2	3	
	IV	1	0	
Mechanism of trauma	Blunt	19	5	1
	Penetrating	0	1	
Pre-operative vital signs	HR (bpm) average	87	84	0.357
	Median	80	80	
	Range	60–130	70–100	
	SPB (mmHg) average	118	115	0.396
	Median	120	120	
	Range	70–150	95–140	
	GCS 15	17	6	1
	14	2	0	
	13	0	0	
	ATLS class I	10	4	0.669
	II-responder	8	2	
	III-responder	1	0	
Pre-operative blood test	BE (mmol/L) average	− 0.9	3.5	**0.036**
	Median	− 1.1	3.2	
	Range	− 7–5	− 2.3–9.7	
	Lactate (mmol/L) average	1.5	1.1	0.245
	Median	1.4	0.7	
	Range	0.5–4.4	0.6–1.9	
	HB (g/dL) average	11.8	11.1	0.306
	Median	11.8	10.9	
	Range	15.4–5.3	8.7–14	
ISS	Average	15	11	0.070
	Range	4–26	1–16	
Timing	Early	14	3	0.344
	Delayed	5	3	
Failed NOM		6	2	0.935
AAST grade	I	1	1	0.838
	II	2	1	
	III	4	2	
	IV	10	2	
	V	2	0	
Operative time (mins)	Average	59	135	**< 0.00001**
	Median	54	135	
	Range	28–106	83–193	
Blood trasfusions	Intre-operative	8	0	0.129
	Post operative	4	2	0.606
Intensive care unit	Admission	10	1	0.180
	Average length of stay (days)	1.3	3	0.329
Bowel function recovery (average POD)	NGT removal	2.5	0.8	**0.002**
	Feeding	3.5	1.3	**0.0005**
	Flatus	3.5	3	0.224
	Stool	5.5	3.7	**0.009**
Morbidity	Overall	11	3	1
	Re-operation	1	0	1
	SSI	3	0	0.554
Mortality		0	0	1
Overall length of stay (days)	Average	9	7	0.130
Follow-up	Incisional hernia	3	3	0.125
	Bowel obstruction	1	0	1

**Table 7:** Per-protocol analysis between open (OS) and laparoscopic (LS) splenectomy for isolated splenic injuries

Characteristics		OS [18]	LS [5]	*P*
Gender	M	11	4	0.615
	F	8	1	
Age (years)	Average	55	42	0.127
	Median	51	41	
	Range	21–86	24–59	
ASA	I	10	1	0.319
	II	6	2	
	III	2	2	
	IV	1	0	
Mechanism of trauma	Blunt	19	4	1
	Penetrating	0	1	
Pre-operative vital signs	HR (bpm) average	87	88	0.483
	Median	80	88	
	Range	60–130	75–100	
	SPB (mmHg) average	118	114	0.368
	Median	120	110	
	Range	70–150	95–140	1
	GCS 15	17	5	
	14	2	0	
	13	0	0	
	ATLS class I	10	4	0.423
	II-responder	8	1	
	III-responder	1	0	
Pre-operative blood test	BE (mmol/L) average	− 0.9	0.45	0.310
	Median	− 1.1	0.45	
	Range	− 7–5	− 2.3–3.2	
	Lactate (mmol/L) average	1.5	0.65	0.139
	Median	1.4	0.65	
	Range	0.5–4.4	0.6–0.7	
	HB (g/dL) average	11.8	11.6	0.453
	Median	11.8	11.9	
	Range	5.3–15.4	8.7–14	
ISS	Average	15	11	0.109
	Range	4–26	1–16	
Timing	Early	14	3	0.608
	Delayed	5	2	
Failed NOM		6	2	0.722
AAST grade	I	1	1	0.840
	II	2	1	
	III	4	1	
	IV	10	2	
	V	2	0	
Operative time (mins)	Average	59	128	**0.00001**
	Median	54	129	
	Range	28–106	83–193	
Blood trasfusions	Intre-operative	8 [40]	0	0.130
	Post operative	4	1	1
Intensive care unit	Admission	10	1	0.327
	Average length of stay (days)	1.3	3	0.426
Bowel function recovery (average POD)	NGT removal	2.5	1.0	**0.0072**
	Feeding	3.5	1.4	**0.002**
	Flatus	3.5	3.2	0.344
	Stool	5.5	4	**0.031**
Morbidity	Overall	11 (57%)	2 (40%)	0.630
	Re-operation	1	0	1
	SSI	3	0	1
Mortality		0	0	1
Overall length of stay (days)	Average	9	7	0.158
Follow-up	Incisional hernia	3	3	0.079
	Bowel obstruction	1	0	1

## Discussion

The cornerstone of debate about splenectomy in stable high-grade splenic injury (AAST grade III–V) is the role of NOM and AE. Recent meta-analysis by Crichton et al. including 23 studies demonstrated how AE allows for decrease in rate of NOM failure from 45–75% to 9–12% in grade IV–V splenic injuries [26]. Similar results were reported by Requarth et al. in 2011 [27]. However, according to the study by Dolejs et al. on the national trauma database between 2008 and 2014 [28], splenectomy rate seems to be stable despite the increased use of AE regardless of the grade of splenic injury, questioning if this should be the first choice treatment in high-grade stable splenic injuries [3].

Hypotension in the field and on initial presentation to the ED, large volume blood transfusion, altered mental status, age above 55 years, hemoperitoneum volume > 250 ml, higher ISS/AAST-OIS grade, associated abdominal injuries, and splenic vascular abnormalities (pseudoaneurysms, arteriovenous fistulae or contrast blushes) have all been identified as risk factors for NOM failure [28–31]. Similarly, factors including subcapsular hematoma and intra-peritoneal contrast blush on CT scan have been suggested as additional independent risk factors for requiring splenectomy [6, 32]. NOM (± AE) failures after penetrating splenic injuries in stable patients can be particularly common, due to the frequent high grade of splenic trauma and the high rate of diaphragmatic lacerations (up to 60%) and other visceral injuries that require immediate laparotomy [33].

Another critical but often underappreciated concern with AE is the rate of major early and delayed complications, including re-bleeding, splenic infarction, abscess, acute renal insufficiency, ARDS, femoral pseudoaneurysms and other access-site complications [28, 33–41]. Although AE is often touted as a “spleen preserving” adjunct to NOM that helps preserve the innate immune function of the spleen, recent literature is calling this assumption into question. In an analysis of 37,986 patients from the Nationwide Readmissions Database, AE was associated with a significantly increased risk of both early and late infectious complications vs NOM without AE, and even had a higher rate of organ space infection at 1 year compared to patients who underwent splenectomy [42]. Despite the current discussions on the usefulness of AE, the risks and complications related to the procedure, and the risk factors for failure, all hemodynamically non-compromised patients with splenic injury, where NOM with or without AE fails or develops complications can be efficiently managed by LS.

NOM (± AE) for splenic injuries has some clinical, social and economical costs as well that must be taken into consideration. In fact, it requires close monitoring, reliable patient adherence to instructions and strict conduct for several weeks, long-term follow-up to reduce the risk of delayed rupture, as well as repeated evaluations or imaging to rule-out possible complications (e.g., pseudoaneurysms) [10, 43]. Therefore, not all patients may be suitable for this kind of management strategy. For instance, fit adult patients or professional athletes may prefer to have the spleen removed instead of facing a long hospital stay and many days of home recovery and strict conduct limitations in order not to lose business commitments. Other examples of patients, where NOM (± AE) may not be indicated are mentally impaired or socially disadvantaged and vulnerable individuals (e.g., homeless).

In addition, general surgeons outside major trauma centers may not feel comfortable in pursuing NOM in higher splenic injury grades and with the spreading of minimally invasive skills some may feel more confident in performing a laparoscopic splenectomy than a high-risk NOM.

Furthermore, to safely undertake NOM (± AE) for high-grade splenic injuries, an operating room and operative team immediately available at all times is required, but this is not the case in many hospitals.

Moreover, there are conditions, where AE fails because of technical difficulties (such as celiac trunk stenosis or tortuous kinking of the splenic artery) which precludes adequate or safe embolization and haemorrhage control. Another major complication of AE is splenic infarction and subsequent conversion to a splenic abscess that requires percutaneous drainage or operative intervention. AE also does not exclude the risk of delayed splenic rupture, and when this occurs there is often no choice but to proceed with emergent open splenectomy.

Some polytrauma patients may require urgent prone spine surgery or prolonged orthopaedic surgery with significant risk of bleeding [6, 43]. In other cases, diaphragmatic injury is associated with splenic trauma and needs immediate repair. In addition, NOM in penetrating splenic injury is rarely indicated. Finally, in patients with splenic injury in pathologic splenomegaly (lymphoma, tropical, autoimmunity, etc.) splenectomy might be considered the treatment of choice. All these examples are good candidates for LS. Other cases are deemed candidate to splenectomy solely based on the trauma surgeon’s evaluation and experience.

Literature on LS for trauma is overall limited, but the published case reports and case-series all suggest that the laparoscopic technique is safe and feasible [14, 17–24]. In 2015 Ermolov et al. published a case series of 23 LS, showing how these patients had better recovering conditions when compared to laparotomy without increased complications or mortality [19]. It was acknowledged that the minimally invasive procedures were more time-consuming. More recently, Shamin et al. reported on the national trauma database (113 laparoscopic splenectomies) demonstrating the safety and feasibility of the procedure in selected cases and experienced hands [15] in terms of comparable mortality, length of stay and major complications, but did not investigate on post-operative bowel recovery. Both these published experiences found similar results to the present study.

Unique from other case series, our current study includes 1 case of penetrating trauma. The results of this study demonstrate how LS in hemodynamically stable trauma patients can be equally indicated in both young and elderly patients, low and high AAST grade splenic injury, low and high ISS score. Compared to OS, LS significantly improves the post-operative recovery with no increased morbidity or mortality. According to these results and in line with the previous published data [14–24], LS seems non-inferior to OS in selected hemodynamically stable trauma patients, where NOM has failed or is deemed not-indicated. However, the presence of both adequate laparoscopic skills and experience in trauma surgery are fundamental to undertake this kind of procedures and achieve good results. The longer operative time of LS has been reported in the other similar publications and is expected to decrease along with improving surgeon’s experience and confidence.

The main strength of the study is the single operator laparoscopic case series that allowed for uniform operative data. Secondly, the high specialization of the institution (level I trauma center), with a dedicated multidisciplinary trauma team (radiologist, intensivist, surgeon) allowed a high standard of trauma care and expertise in dealing with trauma patients. On the other hand, a crucial limitation of the study is the small sample size, which can be explained with patient selection, the novelty of the technique and the low overall volume of abdominal trauma in European countries. The small sample size of our study probably affects the generalization applicability of the results but is proportionate to the national trauma database report by Shamin et al., where 113 LS were performed across the USA in 9 years (12 per year in the whole USA) [15] which is, to the best of our knowledge, the largest single operator case series of LS for trauma. Another limitation is the retrospective analysis that prevented complete reporting on factors such as postoperative pain. Finally, there was inter-operator variability with our laparoscopic experience being solely performed by a single surgeon and our open splenectomy cases having multiple trauma surgeons contribute to our experience.

A potential criticism to a more liberal policy for enlarging the indications to a definitive laparoscopic splenectomy to the patients who are stable but have high-grade splenic injuries (grade III and above ± contrast blushes) is the loss of the spleen and its immunologic and hematologic function, including the theoretical increased risk of overwhelming post-splenectomy infection (OPSI). Nevertheless the immunologic function of the spleen is clinically significant mainly in paediatric patients and young ages. OPSI incidence may be over-estimated and may be very low, even in paediatric populations. In a recent study including 116 children with BSI, 27 underwent splenectomy and 66 patients were treated by a spleen preserving therapy (including embolization) [44]. Only 2 out of 27 splenectomized patients were adequately vaccinated, 5 patients without a spleen used prophylactic antibiotics, and about half of the asplenic patients had adequate knowledge of the risk that asplenia entails. A total of 22/27 splenectomized patients were neither adequately vaccinated nor received prophylactic antibiotics. There was no OPSI seen in this study population during the 1116 follow-up years [10, 44]. Incidence of OPSI in adults is reported in isolated cases than true epidemiological data. The precise incidence of OPSI remains controversial. Overall, the most reliable data related to incidence estimate approximately 1 case occurring per 500 person-years of observation. Asplenic children younger than 5 years, especially infants splenectomized for trauma, may have an infection rate of greater than 10% [43, 45]. Physicians should also be careful not to interpret as an OPSI, the occurrence of severe chest or abdominal infections or other infections or seasonal flu that are NOT or unlikely to be related to splenectomy and would have occurred regardless the patient had their spleen removed.

Finally, laparoscopic splenectomy can easily be combined with autotransplantation of a fragment or single segment of the native spleen, even if this was shattered, and may, therefore, represent a minimally invasive possibility for preserving splenic function after traumatic splenectomy [46, 47]. Laparoscopic Autotransplantation of a fragment of the native spleen after morcellation and extraction of the traumatized spleen, is theoretically worth practicing and likely the subject of a future prospective study.

### Strenghts and limitations of this study

We acknowledge the limitation of the present study and series. The two groups are different and not fully comparable; however, many patients are well selected but the open group is a group of patients when were in any case hemodynamically stable and were potentially candidates to be approached laparoscopically. They represent the best control group for a non randomized comparative study as it is the present study. In fact Randomization is not possible usually in Acute Care setting and probably not feasible at all especially in Trauma setting. The attempt of this study is to demonstrate that lap splenectomy is feasible and safe in Stable and well selected patients with high-grade spleen injuries, and may offer significant advantages over a persistent and often overused NOM + AE (considering all drawbacks and failure rates of NOM + AE in high-grade injuries) and even more significant advantages over an old fashioned open splenectomy which still carries the short and medium-long term morbidity (including postoperative pain, SSI, hernias etc.) intrinsic to the laparotomy.

When deciding the selection criteria for laparoscopic approach, we have analyzed 2 groups in comparison: both groups had a splenic injury of High-Grade AND any indications for a definitive splenectomy (either because NOM ± AE was not feasible or could not achieve definitive control of the contrast blushes/pseudoaneurismys, or because there were ongoing minor venous bleeding from the injured parenchyma and required ongoing Blood transfusions or because they needed major associated orthopedic, e.g., spine surgery and the NOM was deemed not safe to pursue or because they had some other type of relative contraindications to NOM, e.g., travelers, living alone, unreliable compliance to recommendations of rest after NOM etc.). All these patients with high-grade splenic injuries and indications for a splenectomy were all STABLE and could have approached by open or lap splenectomy. We have observed that they were approached open when they went to OR during an On Call day of two of the Trauma Surgeons Attendings in Bologna Hospital, whereas they were managed by laparoscopic splenectomy when they went to OR during the On Call days of the Trauma Surgeon Attending who had MIS advanced expertise. Basically similar group of patients with quite similar injuries AND an indication for removing the spleen without pursuing NOM, ALL Stable and potentially candidate for lap splenectomy, BUT different Days on Call —> 1 group was treated by open and the other group was approached laparoscopically.

Despite the above criteria are not fully meeting the definition of randomization, the assignment of the patients to OS or LS, occurred randomly, and the first (open) group is somehow comparable (and above all patients have the main criterion of hemodynamic stability) to the lap group and could have been likely approached laparoscopically if all Trauma attendings have had an Advanced MIS Expertise as well.

## Conclusions

Laparoscopic splenectomy for trauma patients is gradually gaining acceptance among the scientific community. Due to the variable nature of trauma, only small case series with low scientific evidence exists [48]. Nevertheless, all published data and the present study demonstrates that laparoscopic splenectomy in hemodynamically non-compromized patients with splenic injuries not amenable to NOM (or failed NOM) and adjuncts such as angioembolization is safe and feasible regardless of the age of patients, severity of trauma, or presence of associated injuries. LS is associated with non-inferior morbidity and mortality and significantly improved post-operative recovery compared to standard open splenectomy; however, the potential advantages and safety of minimally invasive surgery must be considered in relation to the level of expertise of the institution, the availability of adequate laparoscopic equipment, and most importantly the presence of an experienced and skilled laparoscopic surgeon. Prospective or randomized controlled trials in patients with hemodynamically non-compromized or ‘quasi-stable’ splenic injuries are needed to better investigate this cutting-edge topic and better characterize the short and long term benefits of laparoscopic splenectomy for trauma.
